# Increased levels of GFAP and purinergic P2X7 receptor in Alzheimer’s disease brain are associated with Aβ, tau pathologies and synaptic loss

**DOI:** 10.1186/s13195-025-01916-2

**Published:** 2025-11-25

**Authors:** Cinzia A. Maschio, Oscar Moreno, Junlong Wang, Upasana Maheshwari, Annika Keller, Uwe Konietzko, Daniel Razansky, Agneta Nordberg, Axel Rominger, Christoph Hock, Jordi Llop, Roger M. Nitsch, Ruiqing Ni

**Affiliations:** 1 Institute for Regenerative Medicine, University of Zurich, Wagistrasse 12, 9th floor, Zurich, 8952 Switzerland; 2 Radiochemistry and Nuclear Imaging Lab, CIC biomaGUNE, Basque Research and Technology Alliance (BRTA), San Sebastián, 20014 Spain; 3 Department of Nuclear Medicine, Inselspital University of Bern, Bern, 3010 Switzerland; 4 Department of Neurosurgery, Clinical Neuroscience Center, University Hospital Zurich, Zurich, 8091 Switzerland; 5 Institute for Biomedical Engineering, University of Zurich & ETH Zurich, Zurich, 8093 Switzerland; 6 Division of Clinical Geriatrics, Center for Alzheimer Research, Department of Neurobiology, Care Sciences and Society, Karolinska Institutet, Stockholm, 14183 Sweden; 7 Neurimmune, Schlieren, 8952 Switzerland

**Keywords:** Alzheimer’s disease, Amyloid-β, Astrogliosis, GFAP, P2X7R, Tau, Synaptic density

## Abstract

Astrogliosis plays an important role in Alzheimer’s disease(AD). We characterized glial fibrillary acidic protein(GFAP) and purinergic P2X7 receptor(P2X7R) alterations in AD and their associations with amyloid-β levels, tau pathology and synaptic density. Immunostaining(P2X7R, GFAP, amyloid-β, and phospho-tau) and autoradiography using [^18^F]JNJ-64413739 for P2X7R were performed in postmortem hippocampal and entorhinal cortex tissues from 42 AD patients and 35 nondemented-controls. Compared with nondemented-controls, AD hippocampi and the entorhinal cortex presented reactive astrocytes with increased GFAP and P2X7R levels. GFAP and P2X7R levels were positively correlated with amyloid-β, CERAD score, phospho-tau, and Braak stage and enriched around cored plaques. In conclusion, we found increased levels of GFAP and P2X7R in hippocampal and entorhinal cortex of AD patients compared with controls, associated with amyloid-β, tau, and Braak stage, highlighting the importance of astrogliosis in AD.

**Supplementary Information:**

The online version contains supplementary material available at 10.1186/s13195-025-01916-2.

## Background

Alzheimer’s disease (AD) is the most common cause of dementia and is pathologically characterized by amyloid-β (Aβ) plaque and tau tangle deposition. Neuroinflammation is considered to play a pivotal role in the pathophysiology of AD. Resident microglia and astrocytes are the main components of the innate immune system and play important roles in AD [1]. Single-cell genomics has revealed disease-specific selective vulnerability and neuroimmune pathways in dementia, chronic inflammatory states and immunosuppression in AD, as well as diverse human astrocyte and microglial transcriptional responses to AD pathologies [2]. Microglial activation visualized by positron emission tomography (PET) has been shown to be correlated with Aβ and tau loads and to propagate jointly with tau across Braak stages and cognitive decline in AD [3, 4]. Astrocyte imaging, such as the use of monoamine oxidase-B tracers and imidazoline binding site 2 tracers, has demonstrated early astrocyte activation in the brains of AD patients and animal models [5–7]. The plasma level of glial fibrillary acidic protein (GFAP) is an early marker of Aβ in AD [8–10]. The serum GFAP level has been shown to be correlated with astrocyte reactivity, brain atrophy and tau pathology [11]. GFAP protein expression has been reported to be upregulated in the hippocampus of AD patients compared to nondemented controls (NCs) [12]. Reactive glia not only associate with Aβ plaques but also parallel tau tangles [13]. However, the links among astrocytosis, amyloid, and tau pathology and synaptic dysfunction are not fully clear.

Purinergic signaling mediated by purinergic receptors, particularly the P2X7 receptor (P2X7R), plays important roles in chronic immune and inflammatory responses [14]. P2X7R is an adenosine triphosphate (ATP)-gated ion channel expressed on astrocytes, microglia and oligodendrocytes in the brain and macrophages in the periphery [14]. P2X7R is expressed at low levels under physiological conditions when the extracellular ATP concentration is below the threshold required for activation, whereas it is activated when ATP levels rise above the threshold under pathological conditions [14, 15]. P2X7R has proinflammatory functions involving the activation of the nucleotide-binding domain, leucine-rich repeat family pyrin domain containing 3 inflammasome; the secretion of proinflammatory cytokines; and the maturation of interleukin-1β [16, 17]. A possible protective role against AD has been reported for the 489 C > T *p2rx7* gene polymorphism [18]. A recent study revealed a significant increase in plasma P2X7R levels in AD patients compared with NCs (sensitivity 90%, specificity 50%) and in patients with mild cognitive impairment [19]. In AD, P2X7Rs are involved in multiple pathological processes, oxidative stress, and chronic neuroinflammation [15]. P2X7R inhibition has been shown to reduce amyloid plaques in animal models of AD by regulating the ability of microglia to phagocytose Aβ and via glycogen synthase kinase-3β and by influencing alpha-secretase-dependent processing [20, 21]. P2X7R inhibition ameliorates the ubiquitin‒proteasome system dysfunction associated with AD [22] and reduces the release of tau-containing exosomes and tau-induced toxicity [23, 24]. Deficiency of the *p2rx7* gene has demonstrated efficacy in enhancing plasticity and cognitive abilities in tauopathy mice and amyloidosis mice [25]. One study showed that P2X7R regulated neuroinflammation in an aged rat model by modulating cytokine and chemokine release [26]. Moreover, P2X7R influences the tau inclusion burden in human tauopathies and induces distinct signaling pathways in microglia and astrocytes [27]. Elevated expression levels of P2X7Rs have been observed in mouse models of AD amyloidosis and in tauopathy mice [25, 28, 29]. These pieces of evidence have implicated P2X7Rs as drug and biomarker targets for AD [30]. P2X7R tracers such as [^11^C]SMW139 [31], [^125^I]1c [32], [^18^F]GSK1482160 [28], [^123^I]TZ6019 [33], and [^18^F]JNJ-64413739 [34] have been developed but not yet reported in AD patients via in vivo imaging or ex vivo evaluation. We recently reported increased uptake of the P2X7R tracer [^18^F]GSK1482160 in vivo in the brains of 7-month-old rTg4510 tau mice, which correlated with [^18^F]APN-1607-measured tau accumulation [28].

Here, we aimed to assess alterations in P2X7R and GFAP expression in postmortem hippocampi and the entorhinal cortex (EC) from NCs and AD patients and to understand the associations of GFAP and P2X7R with Aβ and tau pathologies and synaptic density. Moreover, we evaluated the P2X7R ligand [^18^F]JNJ-64413739 [34] in AD brain tissue.

## Methods

### Postmortem human brain tissue

Forty-two AD patients, each with a clinical diagnosis confirmed by pathological examination, and thirty-five NCs were included in this study (detailed information in Table 1). Paraffin-embedded frontal cortex tissues from autopsies were obtained from the Netherlands Brain Bank (NBB), Netherlands. All materials were collected from donors or from whom written informed consent was obtained for a brain autopsy, and the use of the materials and clinical information for research purposes were obtained by the NBB. The study was conducted according to the principles of the Declaration of Helsinki and subsequent revisions. All the autopsied human brain tissue experiments were carried out in accordance with ethical permission obtained from the regional human ethics committee and the medical ethics committee of the VU Medical Center for NBB tissue. Information on the neuropathological diagnosis of AD (possible, probable, or definite AD) or not AD was obtained from the NBB. Information on the Consortium to Establish a Registry for AD (CERAD), which applies semiquantitative estimates of neuritic plaque density and the Braak score in the presence of neurofibrillary tangles (NFTs), is provided in Table 1. Patients with pathologies other than AD were excluded from the study.

**Table 1:** Demographic and neuropathologic characteristics of the AD patients and NCs

Characteristic	NC (n = 35)	AD (n = 42)	*P *value
Age, mean (SD), y	82.4 (7.1)	82.2 (8.0)	0.91
Sex, No. (%)			
Female	19 (57.1)	35 (83.4)	
Male	15 (42.9)	7 (16.6)	
Braak stage			
0-II	31	4	0.00
III-VI	5	13	
V-VI	0	25	
Amyloid presence			
O	14	0	0.00
A	5	0	
B	6	8	
C	7	28	
Postmortem delay (PMD), mean (SD), h	6.3 (3.6)	4.5 (1.4)	0.01
ApoE ε4 allele status, %, carrier	13.3	61.0	
ApoE ε4 0 allele	26	16	0.01
ApoE ε4 1 allele	4	17	
ApoE ε4 2 allele	0	8	

The values are shown as the mean (standard deviation)

*Abbreviations*: *AD* Alzheimer’s disease

### Immunohistochemistry and Immunofluorescence staining

Paraffin-embedded fixed postmortem human brain tissues (from 35 AD patients and 32 NCs) were cut into 3 μm sections via a Leica microtome (Leica Microsystems, Germany). Hematoxylin and eosin (H&E) staining was performed according to routine procedures for each patient to provide anatomical information and to determine whether there were abnormalities in the brain. GFAP immunohistochemical staining and P2X7R immunofluorescence staining were performed on 35 AD and 32 NC postmortem human brain tissues to visualize the morphology and pattern of the hippocampus. To determine the colocalization of P2X7R with astrocytes and microglia and its association with tau and amyloid pathology, triple staining was performed on 6 AD and 4 NC hippocampal and EC tissue slices. In addition, we performed triple staining in the hippocampus and EC of 3 AD, 3 NC via antibodies against P2X7R, anti-calcium-binding adapter 1 (Iba1), purinergic 2Y12 receptor (P2Y12R), transmembrane protein 119 (TMEM119), and cluster of differentiation 68 (CD68). CD68 is a marker for phagocytic microglia, whereas P2Y12R is a marker for homeostatic microglia and TMEM119 staining Anti-phospho-Tau (Ser202/Thr205, AT-8) to highlight hyperphosphorylated tau typical for tau pathology, anti-Aβ_1− 16_ (6E10), anti-Aβ_17− 24_ (4G8) showing amyloid pathology were used [35, 36]. Antigen retrieval of the paraffin-embedded fixed human brain tissue sections was performed with citrate buffer. First, the sections were heated in buffer for seven minutes at 700 W, followed by 20 min at 280 W. Last, the sections were kept at room temperature without a cover for 20 min. After being blocked in 5% donkey serum in 1% Triton-phosphate-buffered saline (PBS), the sections were incubated with primary antibodies including AT-8, 6E10, P2X7R, GFAP, P2Y12R, CD68 and TMEM119 overnight at 4 °C with mild shaking. For immunofluorescence staining, on second day, the samples were incubated with their corresponding secondary antibodies for two hours at room temperature. The sections were incubated for 15 min with 4′,6-diamidino-2-fenylindol (DAPI), washed two times for 10 min with PBS, and cover slipped after VECTASHIELD Vibrance Antifade Mounting Media (Vector Laboratories, Z J0215) was added (STable 1). For immunohistochemical staining, the sections were then rinsed three times for 10 min each in 0.1 M PBS before being incubated with a biotinylated anti-mouse secondary antibody (suspended in 0.1 M PBS with 0.5% v/v Triton-X) for 2 h. The sections were then incubated with an avidin-biotin complex–horseradish peroxidase for 1 h at room temperature. The sections were developed with 0.025% 3,3′-diaminobenzidine and 0.05% H_2_O_2_ in triphosphate-buffered saline (TBS, pH 7.4) for 3 min. After being mounted on slides, the sections were dehydrated by an ascending alcohol series of 70%, 90%, and 100% (twice each) and Roticlear^®^ for 2 min. Coverslips were finally mounted with Rotimount^®^ mounting medium.

The H&E-, immunohistochemistry- and immunofluorescence-stained brain sections were imaged at ×20 magnification using an Axio Oberver Z1 slide scanner (Zeiss, Germany) with the same acquisition settings for all brain slices. The immunofluorescence-stained brain sections were also imaged at ×10 and ×63 magnification via a Leica SP8 confocal microscope (Leica, Germany).

### Image analysis

For the human hippocampus, manual delineation of the adult human brain based on the Allen atlas was performed via Qupath and ImageJ (NIH, U.S.A.). The cornu ammonis (CA)1, CA2/3, dentate gyrus (DG), subiculum (Sub), EC, and parahippocampal gyrus (PHG) were drawn. GFAP coverage was quantified with ImageJ (NIH, U.S.A.). The slide-canned image was color-deconvoluted, followed by the moment autothreshold on the 3,3’-diaminobenzidine (DAB) channel to obtain a binary image. Region of interest (ROI)-based analysis revealed the % area covered by the antibody by subtracting the obtained background value of the % area from 100. Normalization was performed by dividing all values by the normalization factor and calculating the average values in the CA1 of the NCs.

P2X7R fluorescence intensity was measured in the hippocampal subregions and the EC. The mean intensity was calibrated with the background intensity of each scan. A z-project with maximum intensity was generated from the image acquired by using a slide scanner, following the HiLo lookup table in ImageJ (NIH, U.S.A.). Finally, the mean fluorescence signal of the background was subtracted from the mean fluorescence of the image to obtain the fluorescence intensity of P2X7R.

For analysis of the association between P2X7R and Aβ plaques, three regions were chosen for the slide scanner images. Fifty micrometres from the inside of the cored and diffuse Aβ plaque was defined as the periplaque region [37]^,^ and another 70 μm away from the periplaque region was considered the parenchymal P2X7R. In ImageJ, P2X7R fluorescence intensity and % area were measured after an appropriate threshold was chosen (*n* = 3, 15 cored plaques and 13 diffuse plaques). To avoid bias from heterogeneity, each value was normalized by dividing it by the corresponding parenchymal value. To compute the colocalization of P2X7R with astrocytes (P2X7R/GFAP) and with microglia (P2X7R/Iba1 or P2X7R/TMEM119 or P2X7R/CD68, P2X7R/P2Y12R), a threshold was applied to remove the background signal, and a binary image was created. After the channels of interest are merged, the image is converted into an RGB color image, followed by the implementation of the color threshold. The resulting mask yielded colocalization between the two channels, which was quantified as the colocalizing area.

### Autoradiography with [^18^F]JNJ-64413739

The radiosynthesis of [^18^F]JNJ-64413739 is described in detail in the supplementary file [38]. Brain sections from 7 AD patients and 3 NCs were used for in vitro autoradiography [^18^F]JNJ-64413739 to study the regional distribution of P2X7R. Slices were thawed, dried and preincubated for 15 min with Tris-HCl buffer (50 mM, pH 7.4, supplemented with 1 mM MgCl_2_, 1 mM CaCl_2_, 2 mM KCl and 1% bovine serum albumin) at room temperature. The slices were subsequently incubated in Tris-HCl buffer (50 mM, pH 7.4) containing [^18^F]JNJ-64413739 (13.3 nM) for 30 min at room temperature. For the determination of nonspecific binding, the slices were additionally incubated in a 10 µM solution of the corresponding nonlabelled reference compound. All slices were incubated and exposed simultaneously, allowing direct comparison. After incubation, the slices were removed from the bath, washed for 10 min in ice-cold buffer (50 mM Tris-HCl, pH 7.4, 4 °C) and dipped once in ice-cold ultrapure water. After drying over a heating plate (1 min, 40 °C), the slices were exposed to a phosphor sensitive plate for 5 min and subsequently scanned in a phosphor imager (Amersham Typhoon 5, GE, USA) at the highest resolution (10 μm). For image quantification, regions of interest (CA1, CA3, Sub, DG, EC, and WM) were manually delineated on each slice via ImageJ software (NIH, U.S.A.), with immunohistochemistry images serving as a reference. Pixel intensity values corrected to the area of the region were obtained and used for comparative purposes. The percentage of self-blocking (homologous blocking) was calculated as [Total (No blocking)- Nonspecific (+ blocking)/Total (No blocking)] × 100, drawing a ROI in the whole slice of both blocked and nonblocked tissue.

### Mediation analysis

We analyzed different mediation models to test whether associations exist between pathological markers (AT8 and 4G8), glial activation markers (e.g., GFAP and P2X7R) and outcome variable (Y) synaptic proteins (SV2A and SYP). The data from SV2A and SYP was derived from our recent study [39]. A series of regression-based mediation models were applied, following the classical three-path approach: (1) the effect of the independent variable (X) on the mediator (M; path a), (2) the effect of the mediator on the outcome (Y; path b), and (3) the direct effect of X on Y (path c′). The total effect of X on Y (path c) and the indirect effect (a×b) were estimated (STable 2). Bootstrapping with 5000 resamples was used to compute confidence intervals and significance levels of indirect effects. All variables were z scored prior to analysis to enable comparison of regression coefficients. Only z scored variables were used for standardized effect size estimation. Statistical significance refers to the indirect effect (a×b) with *p* < 0.05.

### Statistics

GraphPad Prism (GraphPad v10) was used for statistical analysis. The normality of the data in the AD and NC cohorts was analyzed via the Shapiro-Wilk test. The nonparametric Mann‒Whitney test was used to compare multiple groups. Braak stages (stage 0, I, II, III-IV, and V-VI), comparisons of Aβ levels (O-C converted to 0–4), and comparisons between males and females and between different hippocampal subregions and the EC. Nonparametric Spearman’s rank correlation analysis was performed to assess the correlations between age, CERAD Braak stage, amyloid and tau levels and P2X7R/GFAP levels.

## Results

### Demographics and pathological description

The ages of the individuals in the NC group (82.4 ± 7.1 [67–99], *n* = 35) were comparable to those in the AD group (82.2 ± 8.0 [64–98], *n* = 42) and followed a normal distribution (Shapiro‒Wilk test). A greater percentage of females was noted in the AD group (83.4%) than in the NC group (57.1%). The prevalence of apolipoprotein E (APOE) ε4 carriers was significantly greater in the AD group (61.0%) than in the NC group (13.3%). No abnormalities were observed in the brain tissue slices from the AD or NCs based on H&E staining results. All the AD patients had an amyloid level of B or C and a varying Braak stage (II-VI). There are NCs that exhibit moderate Aβ or tau pathology in the brain on the basis of amyloid and Braak stage scores [40]. Braak stage and CERAD amyloid level information are described in Table 1.

### Elevated GFAP levels in the hippocampus and EC of AD patients and controls

**Fig. 1:**
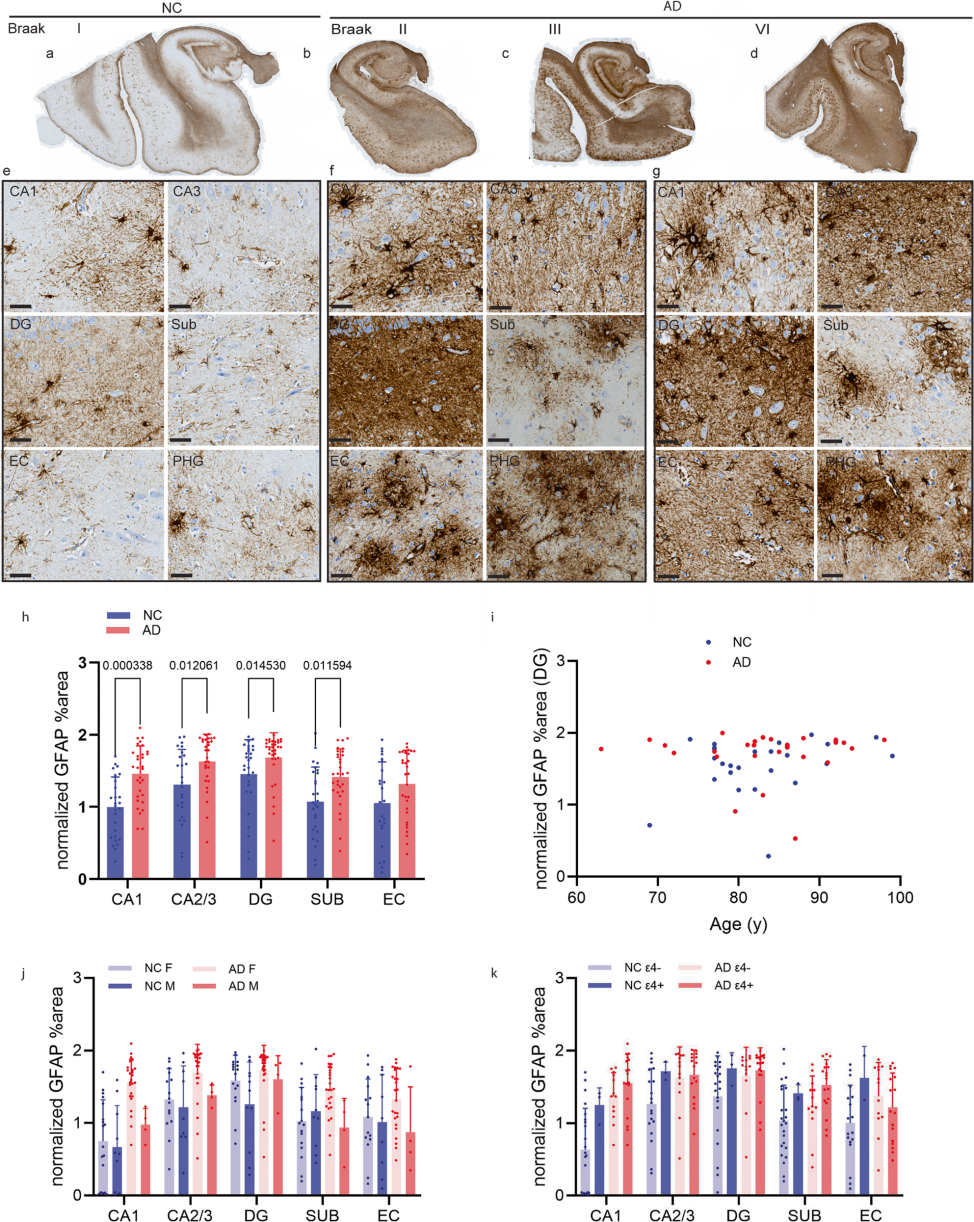
Increased GFAP levels in the hippocampi of AD patients compared with nondemented control individuals. Representative (**a**-**g**) GFAP (brown) immunohistochemical staining of the hippocampus of nondemented control patients (NC #04–015) and AD patients (#99–127, #97–027, #93–12) at different Braak stages. Zoomed-in views of GFAP (**e**-**g**) in the CA1-3 region, dentate gyrus (DG), subiculum (Sub), entorhinal cortex (EC) and parahippocampal gyrus (PHG) (NC #04–015, AD #99–127, #93 − 012). Scale bar = 50 μm (e-g). **h** Increased GFAP coverage in the CA1, CA2/3, DG and Sub regions in AD patients compared with nondemented controls (*n* = 34, *n* = 28). **i** There was no statistically significant correlation between the percentage of GFAP-positive area in the DG and age in the AD and NC groups (*n* = 30, *n* = 25). **j** No difference in GFAP coverage was detected between male and female NCs (*n* = 11, *n* = 18) or between male and female AD patients (*n* = 5, *n* = 28). **k** Apolipoprotein E (APOE) ε4 carriers (NC = 3, AD = 13) and noncarriers (NC = 24, AD = 13) of NCs and AD patients presented similar GFAP expression in the hippocampus and entorhinal cortex

To characterize astrocytes in AD and control brains, we first conducted immunohistochemical staining for GFAP in the hippocampi of 35 AD patients and 32 NCs. Compared with those in the NCs, the protoplasmic astrocyte processes in the hippocampus were thicker in the AD patients (Fig. 1g). Compared with that in NCs, the area of GFAP + area in AD patients was significantly greater than that in NCs (Fig. 1h, CA1, *p* = 0.000338; CA2/3, *p* = 0.012061; DG, *p* = 0.014530; Sub, *p* = 0.11594). In addition, the percentage of GFAP-positive area did not correlate with age in the AD or NC group. There seems to be a trend toward a greater GFAP% area in female AD patients and NCs than in their male counterparts (Fig. 1j). APOE is a lipid transporter produced predominantly by astrocytes in the brain and plays an important role in modulating microglial immunometabolism [41]. APOE ε4 is a genetic risk factor for sporadic AD; however, we found that there was no difference in the GFAP % area between APOE ε4 carriers and noncarriers.

### Increased P2X7R levels in the hippocampus and EC of AD patients compared with NCs

**Fig. 2:**
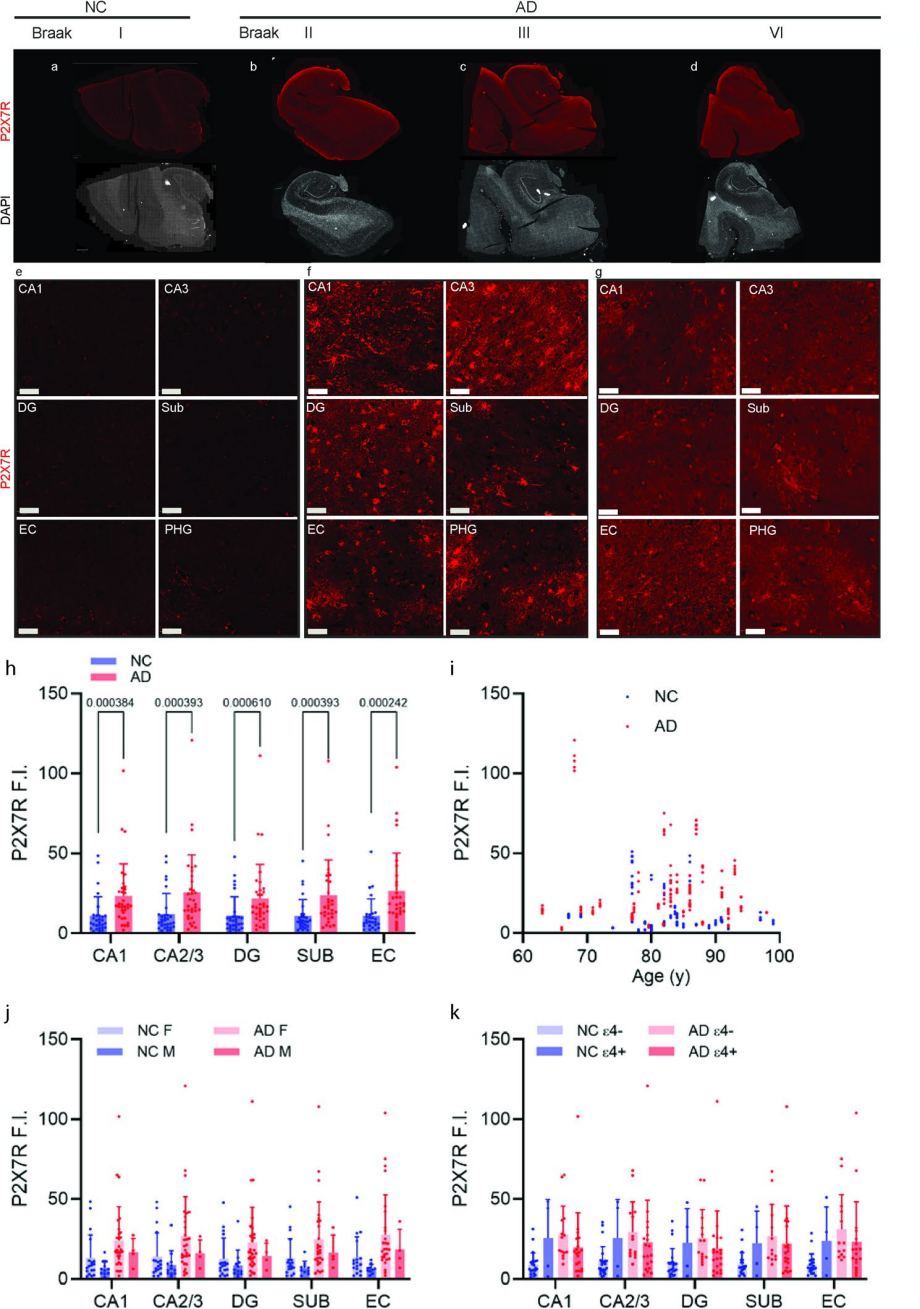
Increased P2X7R levels in the hippocampus and entorhinal cortex of AD patients compared with those in NCs. **a**-**g** Representative P2X7R (red) immunofluorescence staining of the hippocampi of NC and AD patients at different Braak stages. **e**-**g** Zoomed-in views of P2X7R (red). Scale bar = 50 μm (e-g). **h** Increased P2X7R intensity in the hippocampus subfields and the entorhinal cortex of AD patients (*n* = 35) and NCs (*n* = 31). **i** P2X7R expression in the hippocampus and entorhinal cortex did not correlate with age in the AD or NC group. **j** There was no difference in P2X7R expression between male and female NCs (*n* = 13, *n* = 18) or between male and female AD patients (*n* = 5, *n* = 30). **k** There was no difference in P2X7R levels between NC APOE ε4 carriers (*n* = 4) and noncarriers (*n* = 23) or between AD APOE ε4 carriers (*n* = 20) and noncarriers (*n* = 14). CA1‒3, dentate gyrus (DG), subiculum (Sub), entorhinal cortex (EC) and parahippocampus (PHG)

Next, we quantified P2X7R expression in subfields of the hippocampus and in the EC of 35 AD patients and 32 NCs via immunofluorescence staining. The P2X7R level was slightly greater in the EC and CA3 than in the CA1, Sub, and DG in both the AD and NC groups (Fig. 2a-g). We detected a significantly greater level of P2X7R (approximately 2×) in all the hippocampal subfields and the EC in the AD patients than in the NCs (Fig. 2h, CA1, *p* = 0.0004; CA3, *p* = 0.0004; DG, *p* = 0.0006; Sub, *p* = 0.0004; EC, *p* = 0.0002). No correlation was detected between age and P2X7R expression in the hippocampal subfields or in the EC in the AD or NC groups (Fig. 2i). No sex differences in hippocampal or entorhinal P2X7R expression were detected between the AD group and the NC group (Fig. 2j) or between APOE ε4 carriers and noncarriers (Fig. 2k).

### Localization of P2X7R on astrocytes and microglia

**Fig. 3:**
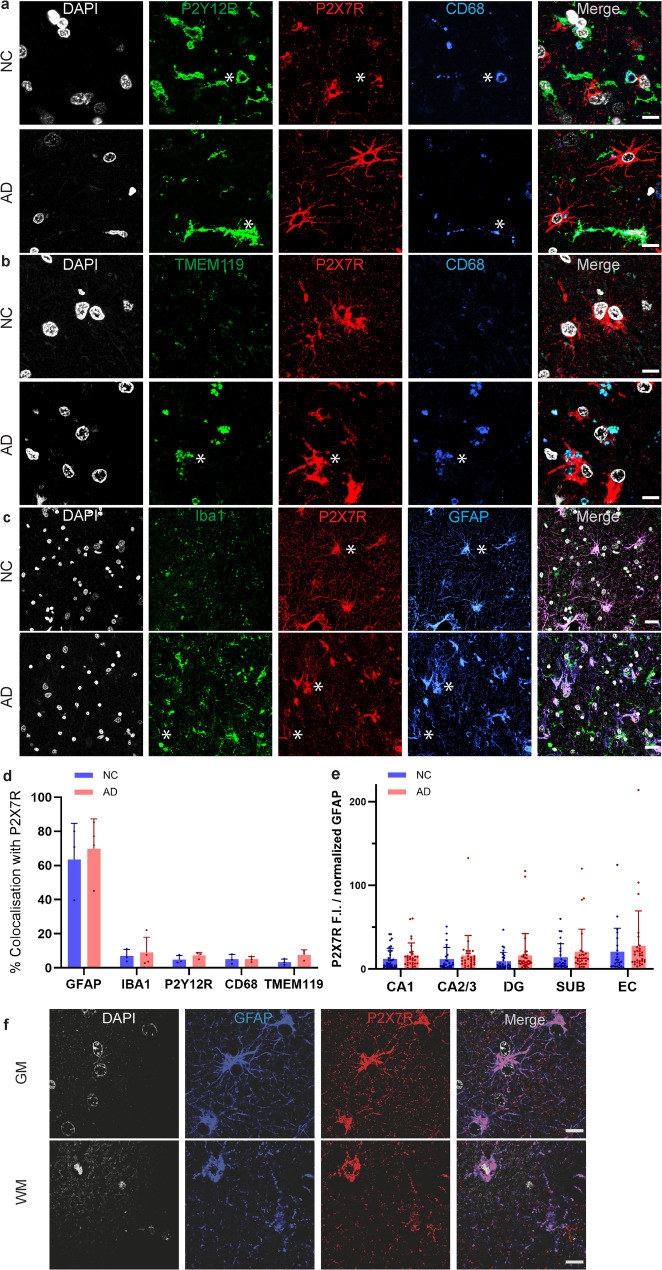
Triple immunofluorescence staining showing the colocalization of microglial P2Y12R with CD68 and TMEM119 with P2X7R and CD68 in the dentate gyrus of postmortem human AD brains. **a** P2X7Rs (red) colocalized with P2Y12R (green) and CD68 (blue). **b** Colocalization in triple staining between TMEM119 (green), P2X7R (red) and CD68 (blue) in the AD subject (*). Scale bar = 10 μm (a, b). **c** P2X7Rs were colocalized mostly with astrocytes (GFAP, blue) and less with microglia (Iba1, green) in both the AD and NC hippocampi (*). Scale bar = 20 μm. Nuclei were counterstained with DAPI (white). **d** Greater colocalization between P2X7R and GFAP (AD: *n* = 4, NC: *n* = 3) than between P2X7R and the microglial markers Iba1 (AD: *n* = 4, NC: *n* = 3), P2Y12R, CD68 and TMEM119 (AD: *n* = 3, NC: *n* = 3). **e** P2X7R expression normalized to GFAP expression did not differ between AD patients and NCs. **f** Immunofluorescence staining of the hippocampus with GFAP (blue) and P2X7R (red) in gray matter (GM) and white matter (WM). Nuclei were counterstained with DAPI (white). Scale bar = 10 μm

Next, we performed confocal microscopy analysis to assess the location of P2X7Rs on astrocytes and microglia in the hippocampus and EC of 3–4 AD and 3 NC. Upon activation, microglia undergo morphological changes from ramified to hyperramified, bushy and finally to amoeboid, where they are fully phagocytic [42]. To determine which subtype of microglia expresses P2X7R, we stained the sections with P2X7R together with a panel of microglial markers. P2X7R was colocalized with the phagocytic marker CD68 (Fig. 3a, b), the homeostatic marker P2Y12R (Fig. 3a), and TMEM119 (a marker for resident microglia with ramified and ameboid morphologies) (Fig. 3b). P2X7R was observed on both Iba1-positive microglia and GFAP-positive astrocytes in the hippocampus (Fig. 3c) and was more strongly associated with GFAP than with microglial markers. Colocalization analysis revealed that in the hippocampus, P2X7R was more prevalent in astrocytes (P2X7R-GFAP overlap: 63% AD, 67% NC) than in microglia (P2X7R-Iba1 overlap: 9% AD, 7% NC; P2X7R-TMEM119 overlap: 7.5% AD, 3.5% NC; P2X7R-P2Y12R overlap: 4.9% AD, 7.2% NC; P2X7R-CD68 overlap: 5.1% AD, 5.0% NC (Fig. 3d, e**)**. This finding is in line with the results of the transcriptomic analysis, which revealed elevated average *P2RX7* gene expression in astrocytes and microglia in the hippocampus and EC of AD patients compared with NCs. Additionally, increased *P2RX7* gene expression was detected in DCLK1 and reactive DPP10 astrocytes and in damage-associated TPT1 microglia in the hippocampus of AD patients (*n* = 26) compared with NCs (*n* = 22) (SFig. 1) (data source [43], details in the supplementary files). P2X7R expression correlated GFAP %area in the CA1 region of AD and NC (*p* = 0.0107, *r* = 0.3312, SFig. 2). Moreover, the density and morphology of fibrous white and protoplasmic astrocytes in the gray matter of the human brain differ [44]. Both fibrous astrocytes and protoplasmic astrocytes were positive for P2X7R (Fig. 3f). Further analysis by confocal microscopy showed that the intensity of P2X7R, GFAP, Iba1 did not differ within the astrocyte or microglia between AD and NC in CA1, CA2/3, DG, Sub and EC (SFig 3). This suggest that the increase of P2X7R fluorescence was likely driven by the increase number of glial cells.

### Positive correlations between GFAP, with Aβ and Tau pathology

**Fig. 4:**
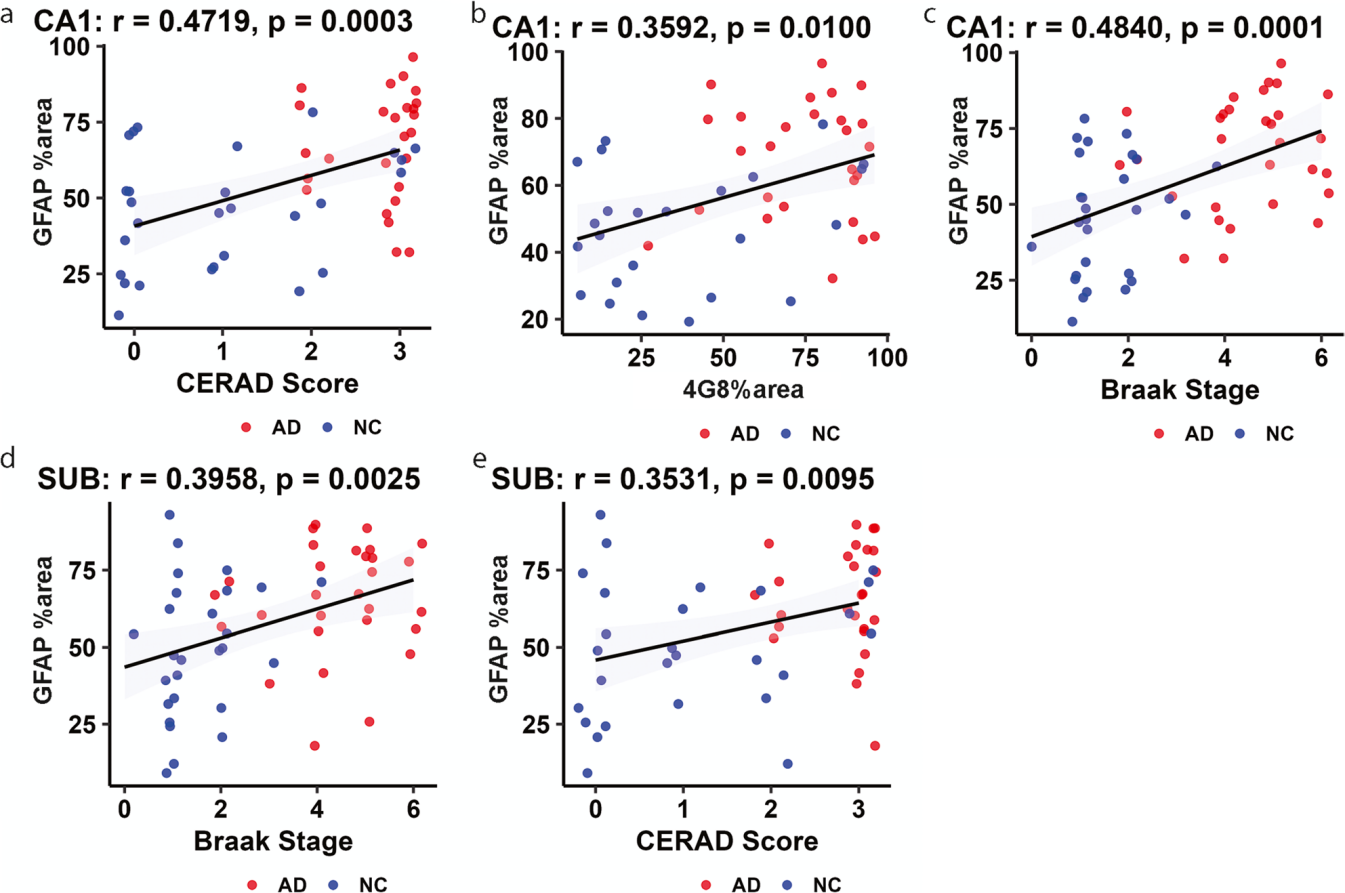
Association between GFAP and p-tau and synaptic density in AD (**a**-**e**) Spearman’s rank correlation analysis between P2X7R and (**a**, **e**) CERAD score, (**b**) 4G8% area, and (**c**, **d**) Braak stage

Next, we assessed the associations between GFAP with Aβ and tau pathologies in 35 AD patients and 32 NC (Fig. 4; SFig. 4**)**. Nonparametric Spearman’s rank correlation analysis revealed that the GFAP level was positively correlated with the CERAD score in AD patients and NCs (*p* = 0.0003, *r* = 0.4719 in CA1; *p* = 0.0049, *r* = 0.3922 in CA2/3; *p* = 0.010, *r* = 0.4292 in the DG; *p* = 0.0095, *r* = 0.3531 in the Sub) (Fig. 4a, e, SFig. 4a, b). GFAP was positively correlated with Aβ levels, as indicated by the 4G8% area in AD patients and NCs (*p* = 0.0100, *r* = 0.3592 in CA1; *p* = 0.0015, *r* = 0.4255 in the DG) (Fig. 4b; SFig. 4c). In addition, the GFAP level was positively correlated with the Braak stage in AD patients and NCs (*p* = 0.0001, *r* = 0.4840 in CA1; *p* = 0.0078, *r* = 0.3651 in CA2/3; *p* = 0.0032, *r* = 0.3780 in the DG; *p* = 0.0025, *r* = 0.3958 in the Sub) (Fig. 4c, d, SFig. 4d, e).

### No mediation effect between GFAP or P2X7R, and pathologies on SYP and SV2A levels

GFAP was positively correlated with SYP% area in AD combined with NCs in the DG (*p* = 0.0040, *r* = 0.4124) and in the EC (*p* = 0.0090, *r* = 0.4128); and with SV2A%area in the EC (*p* = 0.0071, *r* = 0.4030) (SFig. 4f, g, h). However in AD or NC group alone, no correlation was observed between P2X7R, or GFAP with SYP% or SV2A%area. To explore potential intermediary mechanisms linking gliosis with synaptic density in AD, we performed mediation analyses using AT8 (p-Tau), 4G8 (Ab_17 − 24_), GFAP, and P2X7R as candidate mediators and presynaptic markers (SV2A and SYP) as outcomes. The data of SV2A and SYP has been reported in our recent study in which we found that there is a lower level of SV2A (but not SYP) in the hippocampus and EC of AD compared to NCs [39]. We found that no mediation effect between P2X7R or GFAP → AT8 → SV2A or SYP; AT8→ P2X7R or GFAP → SV2A or SYP (STable 2).

### Positive correlations between P2X7R with Aβ and tau pathology

**Fig. 5:**
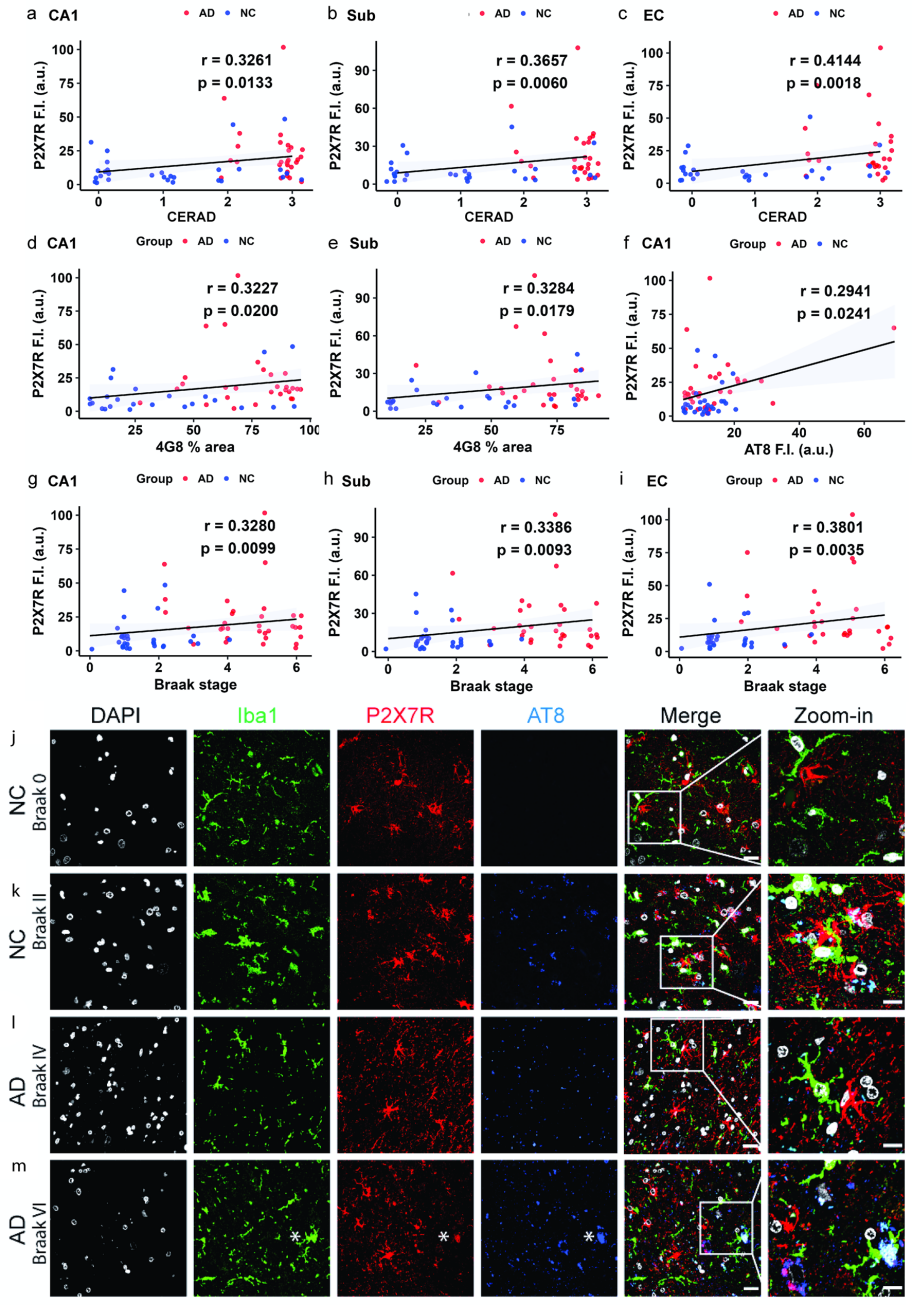
Positive correlation between P2X7R expression and amyloid and p-tau pathologies in the hippocampus and EC. **a**-**i** Spearman’s rank correlation analysis between P2X7R and (**a**-**c**) CERAD score, (**d**-**e**) 4G8% area, (f) AT8 fluorescence intensity (F.I., a.u.) and (**g**-**i**) Braak stage. **j**-**m** Iba1 (green)/P2X7R (red)/AT-8 (blue) staining to determine the association between P2X7R and phospho-Tau. Nuclei were counterstained with DAPI (white). Scale bar = 20 μm. Scale bar of the zoomed-in images = 10 μm

Nonparametric Spearman’s rank correlation analysis was performed in data from 35 AD patients and 32 NCs. P2X7R level was positively correlated with the CERAD score (*p* = 0.0133, *r* = 0.3261 in CA1; *p* = 0.0342, *r* = 0.2786 in CA2/3; *p* = 0.0349, *r* = 0.2752 in the DG; *p* = 0.0060, *r* = 0.3657 in the Sub; *p* = 0.0018, *r* = 0.4144 in the EC) (Fig. 5a-c, SFig. 5a, b). In addition, P2X7R was positively correlated with Aβ levels, as indicated by the 4G8% area in AD patients and NCs (*p* = 0.0200, *r* = 0.3227 in CA1; *p* = 0.0336, *r* = 0.2877 in the DG; *p* = 0.0179, *r* = 0.3284 in the Sub) (Fig. 5d, e; SFig. 5c). P2X7R levels were also positively correlated with p-tau (AT-8) levels in AD patients and NCs (*p* = 0.0241, *r* = 0.2941 in CA1; *p* = 0.0347, *r* = 0.2737 in CA2/3; *p* = 0.0416, *r* = 0.2620 in the DG) (Fig. 5f, SFig. 5d, e). In addition, the P2X7R level was positively correlated with the Braak stage in AD patients and NCs (*p* = 0.0099, *r* = 0.3280 in CA1; *p* = 0.0303, *r* = 0.2753 in CA2/3; *p* = 0.0329, *r* = 0.2691 in the DG; *p* = 0.0093, *r* = 0.3386 in the Sub; *p* = 0.0035, *r* = 0.3801 in the EC) (Fig. 5g-i, SFig. 5f, g).

**Fig. 6:**
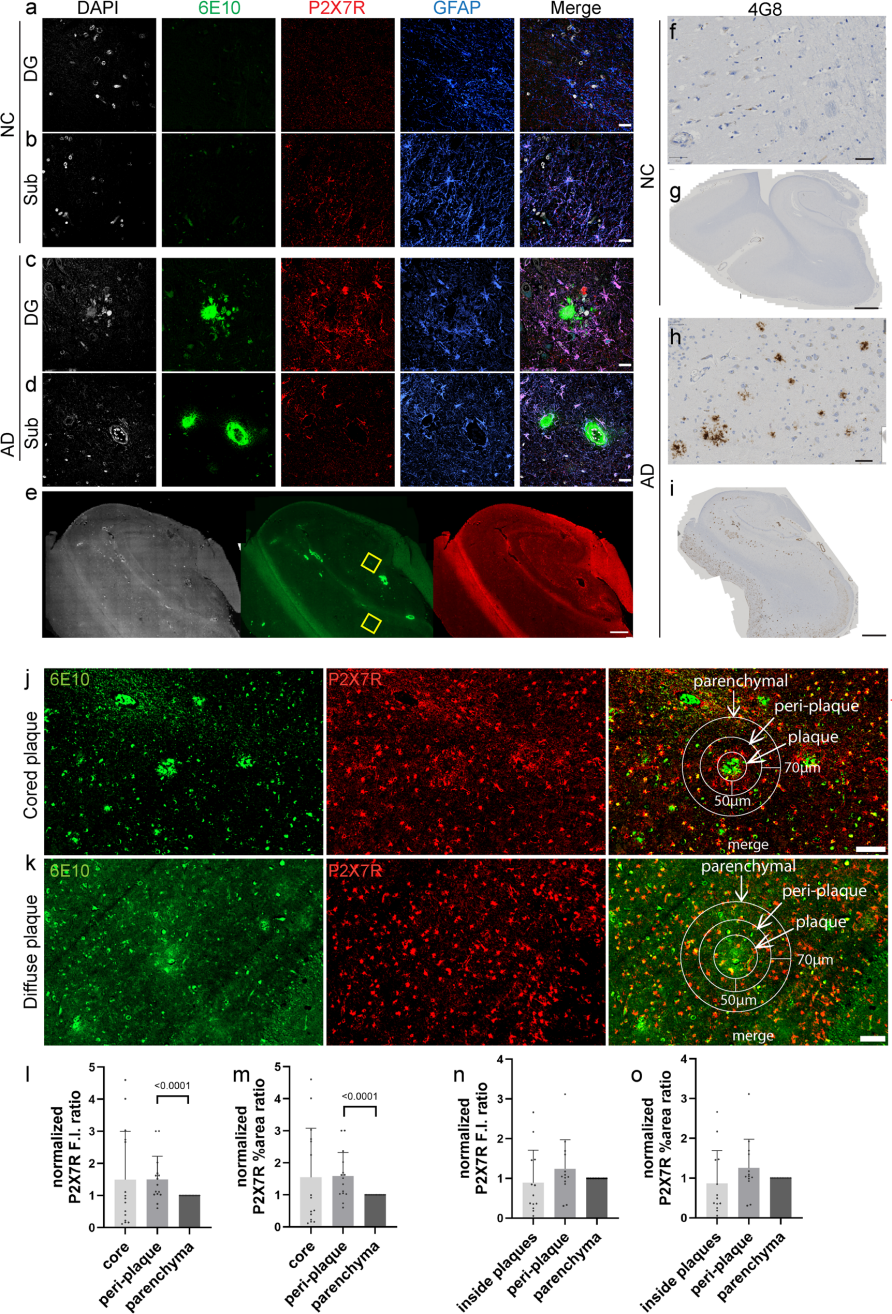
Increased P2X7R with Aβ deposits in the hippocampus and entorhinal cortex of AD patients compared with NCs. **a**-**e** Brain tissue sections from NCs and AD patients were stained for 6E10 (green)/P2X7 (red)/GFAP (blue). **a**-**d** Zoomed-in view of the dentate gyrus (DG) and subiculum (Sub). Increased P2X7R was detected surrounding parenchymal Aβ plaques as well as cerebral amyloid angiopathy (CAA) in the vessel wall. The yellow square in e indicates the location of the zoom in (**c**, **d**). Nuclei were counterstained with DAPI (white). Scale bar = 20 μm (a-d), 800 μm (e). **f**-**i** Brightfield staining revealed amyloid deposits in brown with the 4G8 antibody, indicating Aβ deposition in the AD subject (**h**). Scale bar = 100 μm (**f**, **h**), 1 mm (**g**, **i**). **j**-**o** Regional distribution of P2X7R near cored plaques (**j**) and diffuse plaques (**k**) stained with 6E10. **l**-**o** Increased P2X7R fluorescence intensity and % area in the peri-compact plaque region compared with the parenchymal region (3 patients, 15 plaques) but no difference in the peri-diffuse plaque region (3 patients, 13 plaques). P2X7R (red) 6E10 (green). Scale bar = 100 μm

We further assessed the spatial associations of P2X7R-positive glia with tau deposits and Aβ plaques (in 3 AD and 3 NC). Increased P2X7R levels and GFAP-positive astrocytes were observed around AT-8-positive p-tau inclusions (Fig. 5j-m). In addition, tau-associated microglia [45] also colocalized with P2X7R in the hippocampus of AD patients (Fig. 5m). The identification of tau-associated microglia might indicate susceptibility to neuronal loss [45]. The immunofluorescence reactivity of P2X7R increased around and inside the 6E10-positive parenchymal Aβ plaques (Fig. 6a-c) and cerebral amyloid angiopathy (Fig. 6d). Moreover, an increase in P2X7R fluorescence intensity and percentage area in the peri-plaque region compared with the parenchymal region was observed for compact plaques (*p* < 0.001, *p* < 0.001) but not for diffuse plaques (Fig. 6l-o).

### Autoradiography with the P2X7R tracer [^18^F]JNJ-64413739

**Fig. 7:**
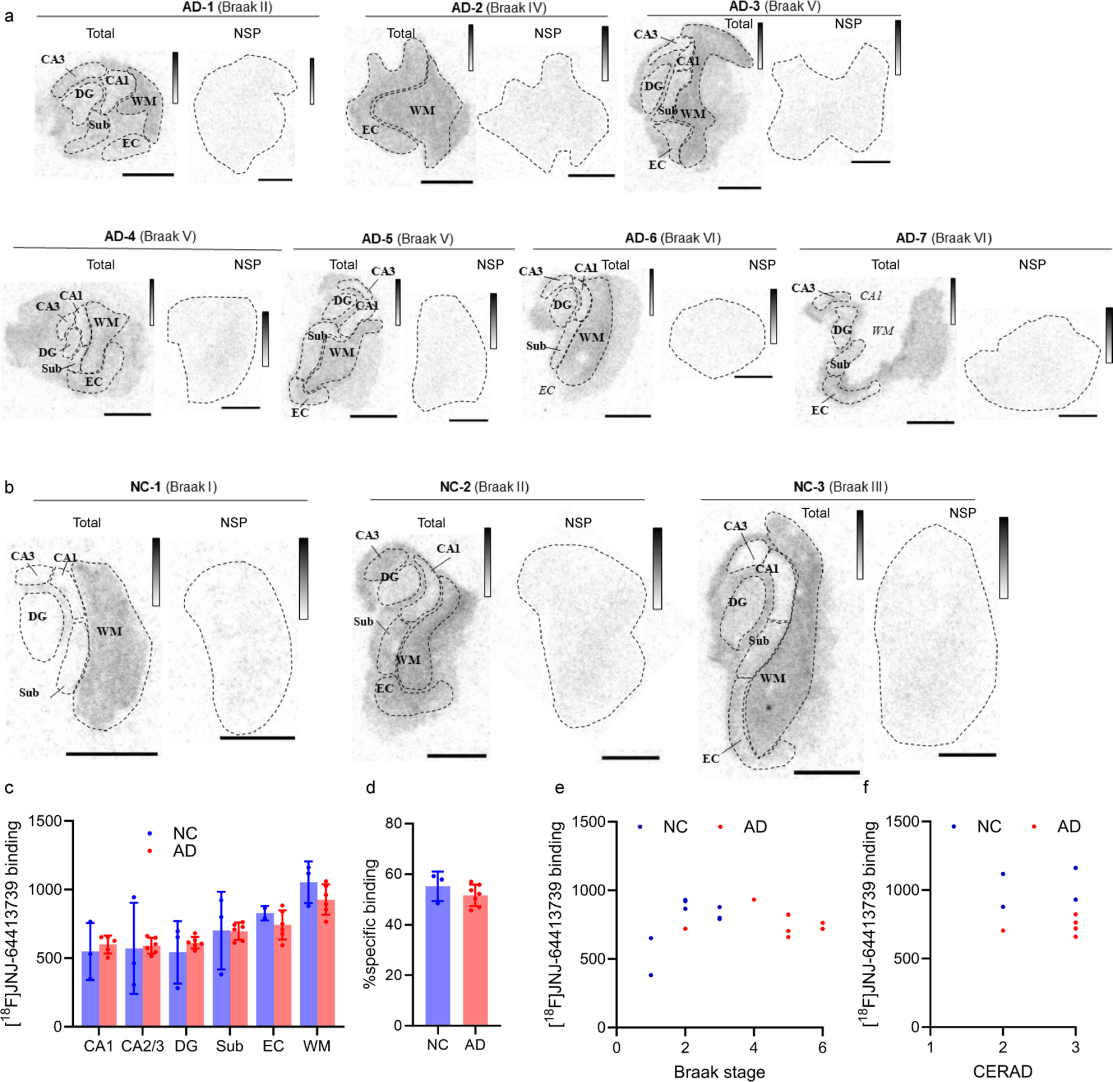
Specific binding of the P2X7R [^18^F]JNJ-64413739 radiotracer results in AD and NC brain sections. **a**,** b** In vitro autoradiography images with [^18^F]JNJ-64413739 in AD and NC hippocampus and entorhinal cortex sections. The corresponding quantification of [^18^F]JNJ-64413739 binding in each subregion is indicated as pixel intensity values. DG = dentate gyrus, Sub = Subiculum, EC = entorhinal cortex, WM = white matter. The gray bars represent 245 (white) to 3352 (black) arbitrary units. Scale bar = 5 mm. **c** Averaged [^18^F]JNJ-64413739 binding (pixel intensity) in each subregion of the hippocampus and EC in AD patients and NCs. **d** Average percentage of specific binding of [^18^F]JNJ-64413739. **e** No association between [^18^F]JNJ-64413739 binding with the Braak stage in AD patients and NCs was detected. **f** Group of [^18^F]JNJ-64413739 binding in AD patients and NCs according to the CERAD amyloid score

Additionally, we assessed the pattern of P2X7R by autoradiography with P2X7R PET tracer [^18^F]JNJ-64413739. Compared with other gray matter regions or hippocampal subregions, [^18^F]JNJ-64413739 presented greater binding in white matter in both the AD and NC groups (Fig. 7a, b). In addition, [^18^F]JNJ-64413739 exhibited a high percentage of specific binding (approximately 51.6% in AD and 55.2% in NCs) in the hippocampal tissue slices (Fig. 7d). However, large variation was observed among both the AD and NC groups. Owing to the limited number of cases, the analysis of regional pixel intensity across Braak stages did not reveal significance (Fig. 7e-f).

## Discussion

Here, we showed increased levels of P2X7R and GFAP in the hippocampus and EC of AD patients compared to NCs, and these levels were positively correlated with Aβ, tau pathology, and Braak stage. The astrocytosis indicated by increases in GFAP levels and morphological changes is in line with the findings of the majority of earlier studies, although prominent atrophic changes in GFAP- and glutamine synthetase-positive astrocytes have also been reported [46–48].

The associations between astrocytosis, Ab and tau pathology in AD have been also investigated in plasma as well as in cerebrospinal fluid (CSF) samples. Clinical studies have supported plasma GFAP as a robust proxy of astrocyte reactivity in the brains of these individuals [49–51], mediating early AD progression [52]. Plasma GFAP levels have been shown to correlate with CSF GFAP levels and have greater accuracy than CSF GFAP levels in discriminating between Aβ–positive and Aβ-negative individuals, even at the preclinical stage [49], and influences the effects of Aβ on tau pathology in preclinical AD [53]. Another study revealed that plasma levels of GFAP are associated with AD tau pathology and with cognitive decline [54, 55]. In contrast, one study showed that plasma GFAP is an early marker of Aβ but not tau pathology in AD [8]. In a recent study, premortem serum GFAP levels were shown to strongly correlate with GFAP- and CD68-immunostained brain areas as well as Aβ and tau tangles in the same patients at postmortem [11]. One of the reasons is the dynamic alterations of astrocytes in AD. In addition, there are isoforms of GFAP that might contribute to this difference [12]. In a recent study, premortem serum GFAP levels were shown to strongly correlate with GFAP- and CD68-immunostained brain areas as well as Aβ and tau tangles in the same patients at postmortem [11].

The relationship between astrocytic activity, synaptic marker as revealed by human imaging, appears multifaceted. Several studies indicate an inverse relationship; for example, elevated plasma GFAP levels are associated negatively with synaptic density (by SV2A PET) [56] and have been shown to mediate the link between tau accumulation and synapse loss in the medial temporal lobe [57]. In contrast, Rohden et al. found positive correlations between CSF GFAP and synaptic regulatory proteins, proposing that astrocyte reactivity may be a distinct phenomenon contributing to synaptic dysfunction, while microglial effects are more dependent on Aβ pathology [58]. Here we did not observe a mediation effect of GFAP or P2X7R with Ab or pTau on SV2A/SYP reduction. This complexity may be partly explained by the biology and complexity in synaptic markers. Recent study using puncta staining has shown that SV2A is expressed only in a subset of synapses and represents a sub-fraction of the total synaptic puncta in the human brain, and overlap partially with SYP + puncta [59]. Positive association between SV2A and postsynaptic marker metabotropic glutamate receptor 5 (mGluR5) by PET have been reported especially in hippocampus and EC [60, 61] and in the medial temporal lobe of AD [62]. Moreover earlier studies showed that hippocampal SV2A level (by PET) negatively correlated with entorhinal cortical tau deposition (by PET) in cognitively normal controls and in early AD [63], and with gray matter microstructure assessed by diffusion tensor imaging [64]. Similar negative association have been reported for mGluR5 (by PET) and tau pathology in the medial temporal lobe in early AD [65]. In addition, studies have shown that increased astroglial reactivity (GFAP) and microglial triggering receptor expressed on myeloid cells 2 contribute to synaptic dysfunction [58]. AD patient-derived high-molecular-weight tau has been shown to impair bursting in hippocampal neurons [66]. Emerging evidence suggests that both microglia and astrocytes actively participate in synaptic remodeling through synaptic engulfment mechanisms. Additionally, the elimination of tau oligomer-containing synapses by microglia and astrocytes in AD has been demonstrated, indicating the importance of glial cells in AD [67]. Astrocytes have been shown to phagocytose synaptic elements through phagocytic receptors including MEGF10 and MERTK [68]. Additionally, astrocytic ephrin receptor A4 signaling is involved in the elimination of excitatory synapses in AD [69]. Complement C1q-dependent excitatory and inhibitory synapse elimination by astrocytes and microglia in AD mouse models has been reported [70, 71]. Another study showed that microglia-synapse engulfment might ameliorate neuronal hyperactivity in AD models [72]. Further studies on the association between astrocytic activity and pre- and post-synaptic dysfunction in AD are needed.

Previous studies on P2X7R levels in the AD brain compared with those in NCs have been inconsistent. Here, we found increased levels of P2X7Rs in the hippocampus and EC in AD patients compared with NC controls. In addition, P2X7R significantly correlated with the level of GFAP in the CA1 region. Future studies are needed to further investigate the subtypes of astrocytes expressing P2X7R. Our findings are in line with those of a previous study showing increased hippocampal P2X7 protein levels via Western blotting and P2X7 mRNA levels in homogenates from 9 AD patients and 6 NCs [23]. Other studies have investigated P2X7R changes only in the cortex: increased expression and activation of P2X7R have been reported in three postmortem studies on the temporal cortex tissue of 5AD vs. 4 NCs and from 6 AD vs. 8 NCs [29, 73]. A recent study revealed that P2X7R levels in temporal cortex tissue were higher only in late Braak-stage AD patients than in early Braak-stage AD patients [27]. Furthermore, immunofluorescence staining revealed the colocalization of P2X7R and GFAP as well as the upregulation of P2X7R in the hippocampus of P301S tau mice [33]. Another study reported no difference in the levels of P2X7R, purinergic receptor P2 × 4R and calcium/calmodulin-dependent protein kinase 2 in pyramidal neurons of the frontal cortex by immunohistochemistry [74]; however, similar to our observations, age- or sex-related differences were not reported in this study [27]. In contrast to the increase in P2X7R revealed by immunofluorescence staining, our autoradiography using the P2X7R tracer [^18^F]JNJ-64413739 revealed a lack of clear differences between AD patients and NCs. Another autoradiography using the P2X7R tracer [^11^C]SMW139 in the temporal cortex reported comparable binding in AD patients and NCs [31]. To date, there has been no in vivo imaging study of P2X7R in AD, and an ex vivo evaluation of P2X7R tracers to determine the utility of P2X7R imaging in AD has been lacking. An earlier study revealed greater binding of the P2X7R tracer [^123^I]TZ6019 in P301S tau mice and [^18^F]GSK1482160 [28] in P301L tau mice than in wild-type mice. Further studies using large sample sizes to evaluate [^18^F]JNJ-64413739 in AD brain tissues are needed.

Our immunostaining results revealed that the P2X7R signal was localized mainly to GFAP-positive astrocytes and was rather limited in microglia in the AD hippocampus. Less than 10% overlap between phagocytic microglia, as indicated by CD68, and homeostatic microglia, as indicated by P2Y12R and TMEM119 staining, with P2X7R, was observed. In addition, transcriptomic data revealed increased P2Y12R and P2X7R gene expression in danger-associated TPT1 microglia and increased P2X7R levels in P2Y12R microglia. An earlier study in rat primary hippocampal cultures revealed that P2X7Rs colocalized with CD68-positive microglia [75]. P2Y12R has also been shown to colocalize with P2X7R in the hippocampus of AD patients and NCs in another previous study [76]. The microglial marker TMEM119 has been shown to colocalize with phagocytic microglia CD68 and Iba1 in resident microglia but not in infiltrating, blood-derived microglia [77, 78]. A recent study showed that TMEM119 binds to Aβ to promote its clearance in 5×FAD mice [79]. The relatively low degree of colocalization between microglia and P2X7R is likely due to the region analyzed, as astrocytes are densely distributed in the hippocampus.

Aβ and tau pathologies have been shown to exert distinct influences and induce disease stage-specific microglial subtypes [45]. Reactive astrocytes acquire neuroprotective as well as deleterious signatures in response to tau and Aβ pathology [80, 81]. An earlier study on postmortem brain tissue revealed that microglial activation does not intensify tau neurofibrillary degeneration in AD brains [82]. Here, the level of P2X7R was positively associated with amyloid and p-tau pathology in the hippocampi of AD patients and HCs. An increased P2X7R% area surrounding compact Aβ plaques but not diffuse plaques in the AD brain was observed. This finding is in line with a recent in situ hybridization-immunohistochemistry study showing that *P2RX7* mRNA is localized to GFAP + astrocytes and CD68 + microglia surrounding Aβ plaques in AD brains [27]. P2X7R protein and mRNA expression is significantly greater in AD patients than in NCs when fetal human microglia are exposed to Aβ_1‒42_ peptides [73]. An earlier study revealed that microglia at advanced and late stages of microgliosis associated with plaques presented increased P2X7R and that P2X7Rs decreased microglial phagocytic activity but enhanced microglial migration to Aβ plaques in J20 mice and P2X7R reporter mice [21]. Mechanistically, P2X7R plays a pivotal role in promoting proinflammatory pathways via Aβ-mediated chemokine release [29]. Aβ blunts the adenosine A2A receptor-mediated control of the interplay between P2X7R- and P2Y1R-mediated calcium responses in astrocytes [83]. In addition, a previous study showed that astrocytic accumulation of tau fibrils isolated from AD brains induces inflammation, neuronal dysfunction and cell-to-cell propagation [84]. Specific P2X7R antagonism in tauopathy models has been shown to reduce the accumulation of tau deposits [85], tau phosphorylation, and tau-induced toxicity [23], indicating that P2X7R antagonism is a potential therapeutic target for AD.

Our study has several limitations. First, the AD group had a greater proportion of females than the NC group did, which may influence the observed effects of glial reactivity on preclinical AD pathology [86]. Second, we did not perform staining for additional astrocytic markers, such as excitatory amino acid transporter 2 and connexin 43, as the analysis of the existing transcriptomics data provided indications of potential alterations. Third, the clinical histories of patients with early- or late-onset AD were unavailable, limiting our ability to assess disease progression. Fourth, the sample size for autoradiography analysis of AD and NC brain tissues was relatively small. Additionally, immunohistochemical staining with stereological analysis is considered the gold standard for quantifying astrocytes and microglia, here we adopted %area and immunofluorescence intensity-based quantification instead. Because postmortem studies capture only a single time point, longitudinal in vivo studies are needed to elucidate how biomarker changes to disease progression. Mechanistic studies, such as glia–neuron co-cultures, can further clarify the relationship between glial activation and synaptic dysfunction under controlled conditions.

In conclusion, we showed that P2X7R expression and GFAP coverage are greater in the hippocampus and EC of AD patients than in NCs and are associated with amyloid and tau pathology, in the AD brain. P2X7R has potential as a promising target for imaging neuroinflammation.

## Supplementary Information

Supplementary Material 1.

## Data Availability

Data is available upon reasonable request to the corresponding author.

## Declarations

Part of the manuscript is shared as preprint on Biorxiv 10.1101/2024.02.28.582443.
