# Supplementary material for: Increased levels of GFAP and purinergic P2X7 receptor in Alzheimer’s disease brain are associated with Aβ, tau pathologies and synaptic loss
Source: Alzheimers Res Ther. 2025 Nov 25;17:274. doi: 10.1186/s13195-025-01916-2 (PMC12751640; doi:10.1186/s13195-025-01916-2)
Supplement: Supplementary file 1 — Supplementary Material 1. [file 13195_2025_1916_MOESM1_ESM.docx]

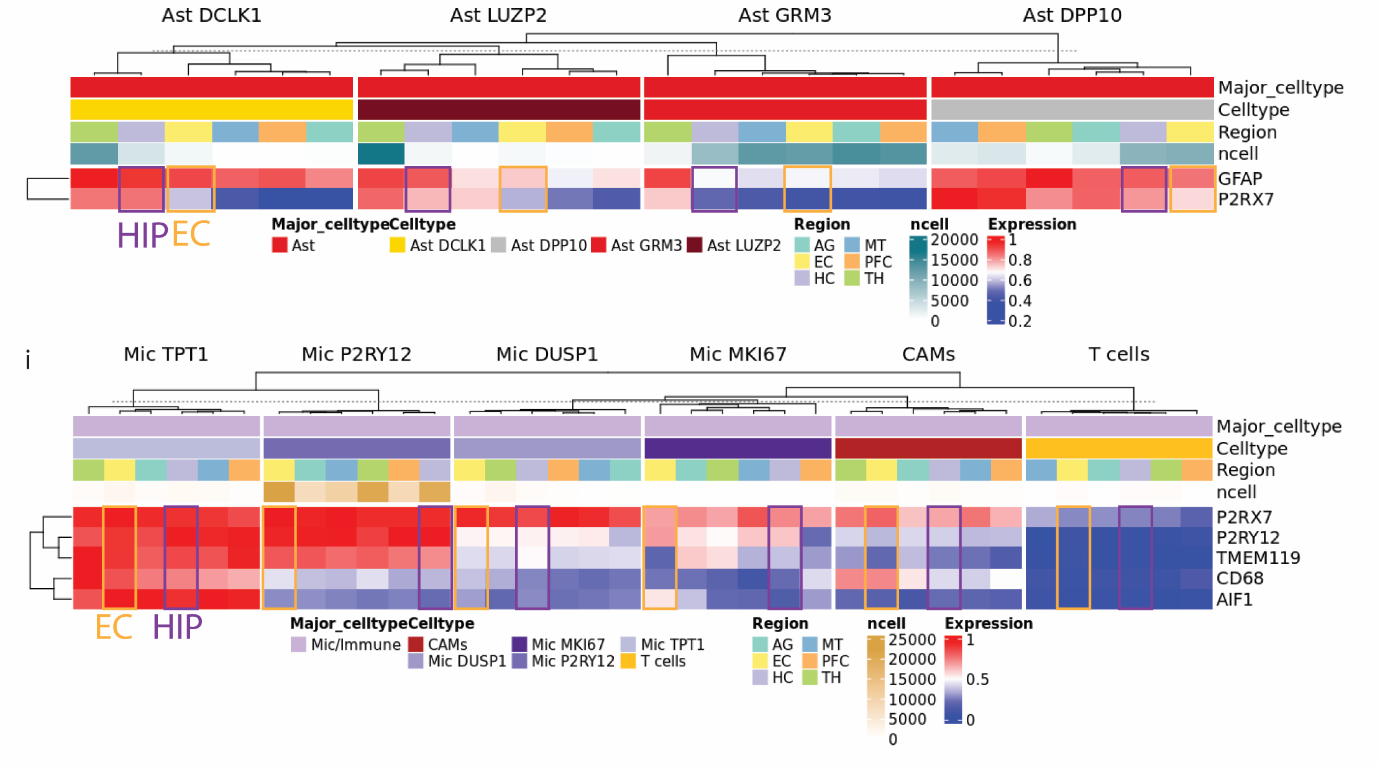


**SFig 1. Heatmap of average gene expression (*P2RX7* and *GFAP*) in subtypes of astrocytes**. Increased hippocampal *P2RX7* and *GFAP* expression was detected in DCLK1 astrocytes and DPP10-reactive astrocytes (data source: <https://ad-multii-region.cells.ucsc.edu>,AD n=26,NC n=22) [2] (**i**) Heatmap of average gene expression (*P2RX7*,*P2RY12,CD68*,*TMEM119*,*AIF*) in microglial subtypes. Increased hippocampal and entorhinal cortical expression of *P2RX7*,*P2RY12*,*CD68*,and *TMEM119* in damage-associated TPT1 microglia. *P2RX7* is increased in surveilled P2YR12 microglia and DUSP1 microglia. <http://compbio.mit.edu/ad_multiregion/>(HIP,HC: purple) (EC: orange)


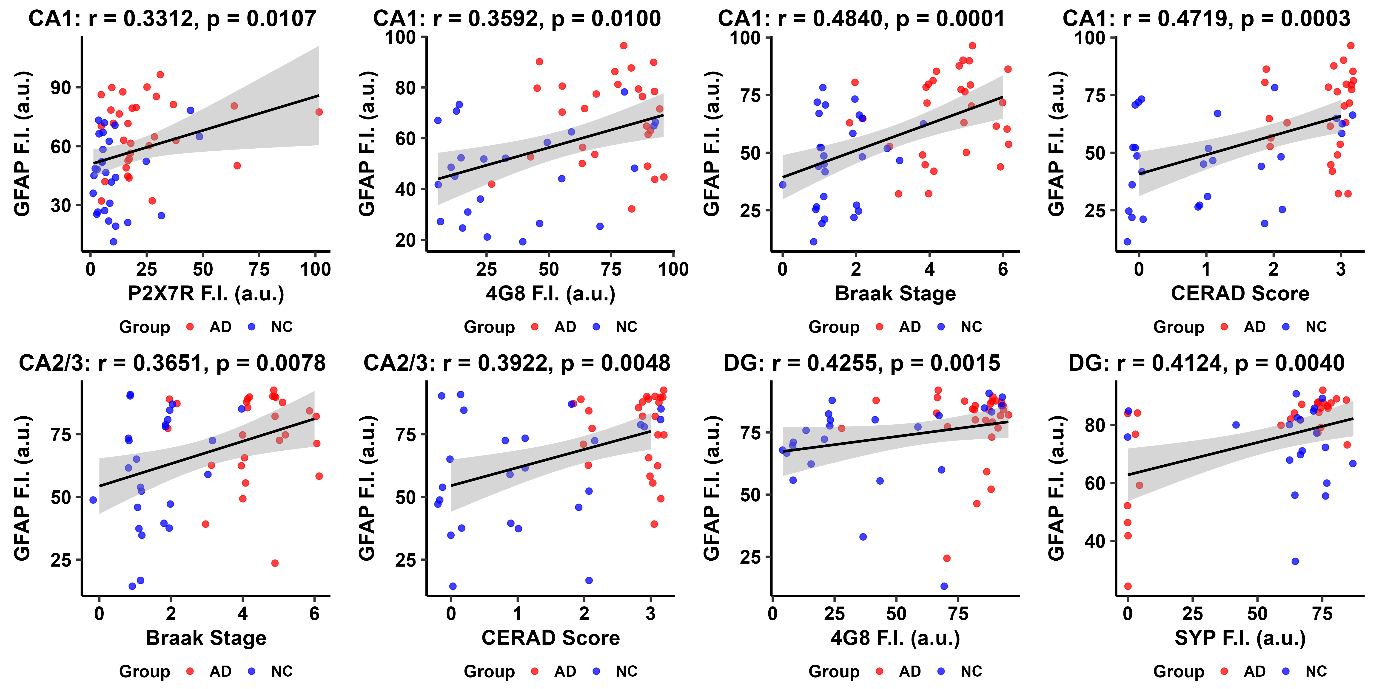


**SFig. 2 Spearman’s rank analysis revealed a positive correlation between P2X7R expression and the GFAP-positive area in the CA1 region of NC and AD patients (combined).**


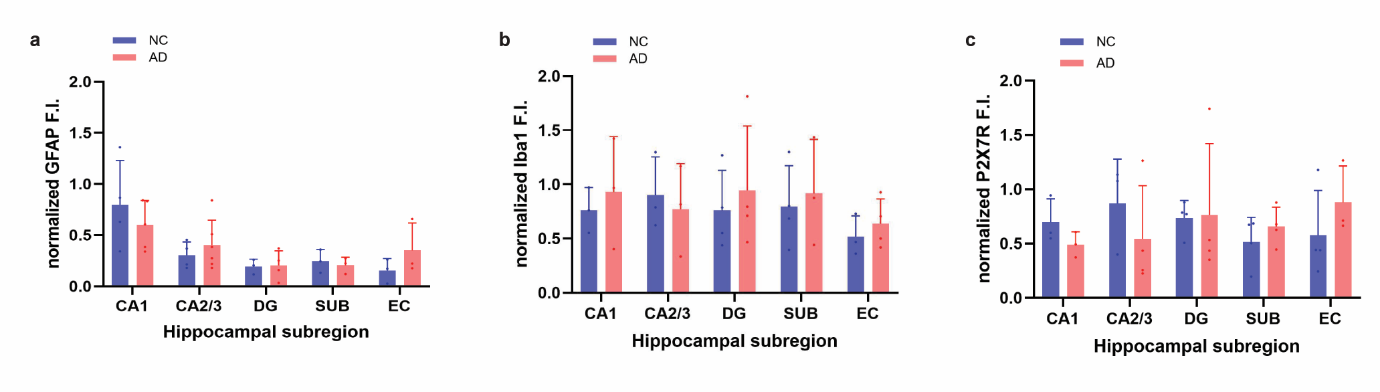


**SFig. 3 Quantification of P2X7R,GFAP and Iba fluorescence intensity by confocal microscopy.** Similar fluorescence intensities (F.I.) of GFAP (**a**),Iba1 (**b**) and P2X7R (**c**) in CA1,CA2/3,dentate gyrus (DG),subiculum (SUB) and entorhinal cortex (EC) were observed between AD patients and nondemented controls (NCs).


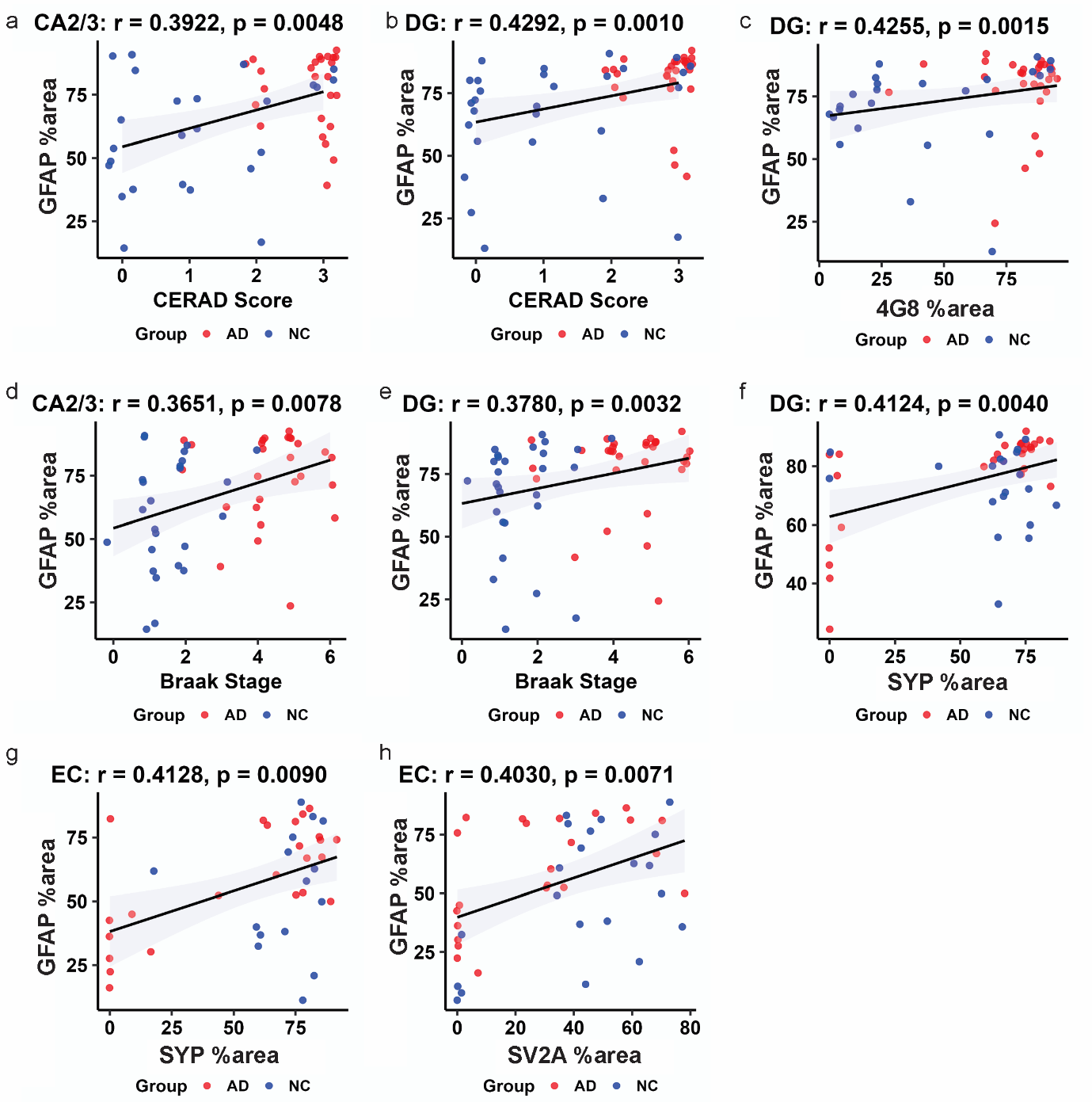
 **SFig. 4 Positive correlations between GFAP and amyloid,Braak stage and SYP in the hippocampus of AD and NC (combined)**. Spearman’s rank correlation analysis between GFAP and (a-b) CERAD scores in the CA2/3 region and DG; (c) 4G8% area in the DG; (d,e) Braak stage in the CA2/3 region and DG; (f,g) SYP% area in the DG and EC; (h) SV2A% area in the EC.


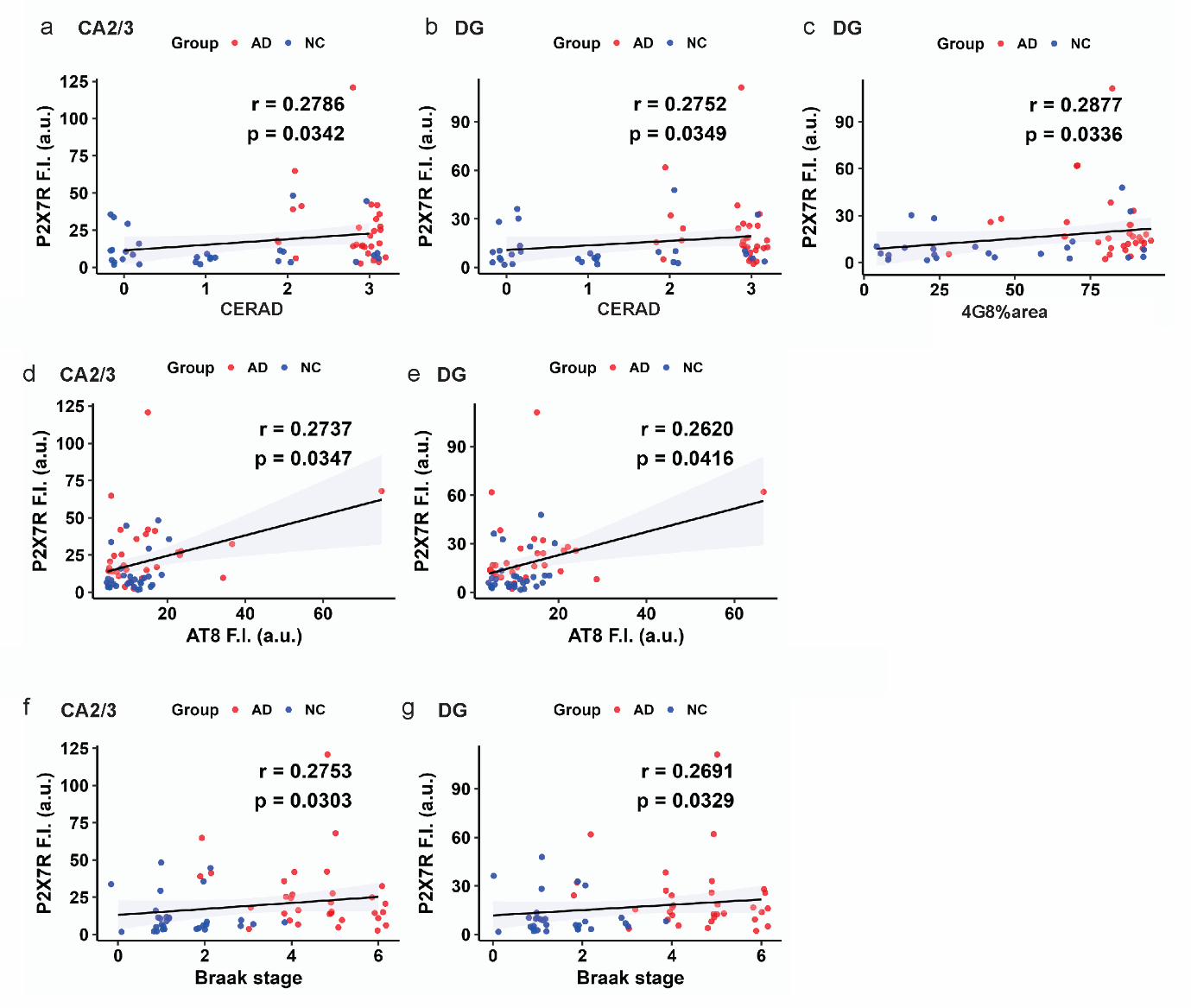
**SFig. 5 Positive correlation between P2X7R and amyloid and tau pathologies in the hippocampus of AD and NC (combined)**. Spearman’s rank correlation analysis between P2X7R and (a-b) CERAD score in the CA2/3 region and DG,(c) 4G8% area in the DG,(d,e) AT8 fluorescence intensity (F.I.,a.u.) in the CA2/3 region and DG,and (f,g) Braak stage in the CA2/3 region and DG.

**STable 1 Antibodies and reagents used**

| **Item** | **Catalog no** | **Dilution** | **Supplier** |
| --- | --- | --- | --- |
| Mouse phosphor-Tau (Ser202,Thr205) monoclonal antibody (AT-8) | MN1020 | 1:1000 | Invitrogen |
| Mouse Purified anti-β-Amyloid,1-16 monoclonal antibody (6E10) | 803001 | 1:1000 | Biolegend |
| Guinea pig GFAP polyclonal antibody | BP5082 | 1:1000 | OriGene |
| Mouse anti-β-Amyloid,17-24 Antibody (4G8) | 800710 | 1:4000 | Biolegend |
| GFAP Monoclonal Antibody (6F2) | MA1-35377 | 1:50 | Thermo Fisher |
| Rabbit ionized calcium-binding adapter molecule 1 (Iba1) | 019-19741 | 1:1000 | WAKO |
| Goat purinergic P2X7 receptor | NBP1-37775 | 1:100 | Novus Biologicals |
| Rabbit purinergic P2Y12 receptor | NBP2-33870 | 1:100 | Novus Biologicals |
| Anti-SV2A antibody (EPR23500-32) | ab254351 | 1:1000 | Abcam |
| Anti-Synaptophysin (27G12) | SYNAP-299-L-CE | 1:1000 | Leica Systems |
| Rabbit transmembrane protein 119 (TMEM119) | HPA051870 | 1:500 | Sigma Aldrige |
| Rabbit recombinant Anti-CD68 antibody [EPR20545] | Ab213363 | 1:1000 | Abcam |
| Alexa fluor 488 donkey anti-rabbit IgG (H+L) | 711-545-152 | 1:500 | Jackson |
| Alexa fluor488 donkey anti-mouse IgG (H+L) | 715-545-151 | 1:500 | Jackson |
| Donkey anti-goat IgG (H+L) cross-adsorbed secondary antibody,Alexa fluor 546 | A-11056 | 1:50 | Invitrogen |
| Alexa fluor647 donkey anti-guinea pig IgG (H+L) | 706-605-148 | 1:500 | Jackson |
| Alexa fluor647 donkey anti-mouse IgG (H+L) | 715-605-151 | 1:500 | Jackson |
| 4',6-Diamidino-2-Phenylindole,Dihydro-chloride | D1306 | 1:1000 | Invitrogen |

**STable 2. Summary of analyses of the effects of glial and pathological markers on SV2A and SYP levels.**

| **X** | **M** | **Y** | **N** | **a path** | | **b path** | | **Indirect a×b** | |
| --- | --- | --- | --- | --- | --- | --- | --- | --- | --- |
|  |  |  |  | **B (p)** | **CI** | **B (p)** | **CI** | **B (p)** | **CI** |
| 4G8 | P2X7R | SV2A | 48 | 0.23 (0.107) | [-0.053,0.522] | -0.23 (0.109) | [-0.517,0.054] | -0.05 (0.246) | [-0.146,0.037] |
| 4G8 | P2X7R | SYP | 44 | 0.22 (0.161) | [-0.090,0.526] | -0.29 (0.072) | [-0.599,0.027] | -0.06 (0.258) | [-0.170,0.046] |
| 4G8 | GFAP | SV2A | 48 | 0.15 (0.322) | [-0.150,0.446] | **0.38 (0.005)** | [0.120,0.637] | 0.06 (0.343) | [-0.060,0.172] |
| 4G8 | GFAP | SYP | 44 | 0.17 (0.282) | [-0.143,0.477] | 0.16 (0.304) | [-0.155,0.484] | 0.03 (0.451) | [-0.044,0.099] |
| AT8 | P2X7R | SV2A | 51 | **0.36 (0.007)** | [0.104,0.619] | -0.15 (0.326) | [-0.463,0.157] | -0.06 (0.349) | [-0.171,0.060] |
| AT8 | GFAP | SYP | 45 | -0.18 (0.23) | [-0.475,0.118] | 0.16 (0.274) | [-0.135,0.462] | -0.03 (0.412) | [-0.099,0.041] |
| AT8 | P2X7R | SYP | 45 | **0.36 (0.014)** | [0.078,0.635] | -0.03 (0.857) | [-0.352,0.293] | -0.01 (0.856) | [-0.122,0.101] |
| GFAP | AT8 | SYP | 45 | -0.2 (0.23) | [-0.519,0.129] | **-0.34 (0.02)** | [-0.627,-0.056] | 0.07 (0.277) | [-0.054,0.187] |
| GFAP | AT8 | SV2A | 51 | -0.18 (0.239) | [-0.483,0.123] | -0.24 (0.08) | [-0.500,0.030] | 0.04 (0.321) | [-0.041,0.126] |
| GFAP | 4G8 | SV2A | 48 | 0.15 (0.322) | [-0.152,0.453] | **-0.53 (0.0002)** | [-0.785,-0.272] | -0.08 (0.33) | [-0.240,0.081] |
| GFAP | 4G8 | SYP | 44 | 0.17 (0.282) | [-0.147,0.492] | -0.2 (0.199) | [-0.519,0.111] | -0.04 (0.402) | [-0.117,0.047] |
| P2X7R | AT8 | SYP | 45 | **0.39 (0.014)** | [0.085,0.700] | **-0.36 (0.023)** | [-0.667,-0.053] | -0.14 (0.081) | [-0.300,0.017] |
| P2X7R | 4G8 | SV2A | 48 | 0.25 (0.107) | [-0.055,0.550] | **-0.42 (0.004)** | [-0.697,-0.140] | -0.1 (0.148) | [-0.244,0.037] |
| P2X7R | AT8 | SV2A | 51 | **0.4 (0.007)** | [0.115,0.686] | -0.23 (0.12) | [-0.526,0.063] | -0.09 (0.168) | [-0.224,0.039] |
| P2X7R | 4G8 | SYP | 44 | 0.22 (0.161) | [-0.092,0.539], | -0.11 (0.461) | [-0.423,0.196] | -0.03 (0.509) | [-0.101,0.050] |

Predictor (X); Mediator (M); Outcome (Y); Each row represents a mediation model testing the indirect effect of a X on Y via a M. Coefficients are reported for the a path (X → M),b path (M → Y),and indirect effect (a×b). All the variables were z scored. The models were adjusted for age and sex; p < 0.05 indicated significance. Indirect effects were estimated via nonparametric bootstrapping (5,000 resamples) with 95% CIs.

**Supplemental methods**

**Transcriptomic analysis**

To determine whether *P2RX7* gene expression is up- or downregulated and to what extent *P2RX7* is expressed on astrocytes and microglia,transcriptomic data were obtained from the Alzheimer’s disease multiregional data repository with age-matched AD patients (n=26,median age 86.6 years) and controls (n=22,median age 86.0) (2). Further information on the sample and methodology are available at (2). The average gene expression was created via the USCS browser (<https://cells.ucsc.edu/?ds=ad-multi-region>) (2) by selecting regions of interest,such as the hippocampus and the entorhinal cortex,the cells of interest (microglia and astrocytes) and the genes of interest (*P2RX7*,*GFAP*,allograft inflammatory factor (*AIF*),*CD68*,*TMEM119*,and *P2RY12*).

**Radiosynthesis of [^18^F]JNJ-64413739**

[^18^F]JNJ-64413739 was prepared in a TRACERlab FX_FN_ synthesis module (GE Healthcare,Waukesha,WI,USA) following a previously described method with minor modifications (1). First,[^18^F]F^−^ was generated in a Cyclone 18/9 cyclotron (IBA,Belgium) via proton irradiation of ^18^O-enriched water via the ^18^O(p,n)^18^F nuclear reaction and trapped on a preconditioned Sep-Pak® Accell Plus QMA Light cartridge (Waters,Milford,MA,USA). Then,[^18^F]F^-^ was eluted from the cartridge with a mixture of aqueous potassium carbonate solution (3.5 mg/0.5 mL) and a solution of Kryptofix 2.2.2. (15.0 mg) in acetonitrile (1.0 mL) and transferred to the reaction vessel. The aqueous [^18^F]F^-^ solution was gradually dried at 60–120°C for 15–30 min under a N_2_ flow to completely remove the water and acetonitrile by azeotropic drying. After complete elimination of the solvent,a solution of the precursor (S)-(3-chloro-2-(trifluoromethyl)pyridin-4-yl)(6-methyl-1-(pyrimidin-2-yl)-1,4,6,7-tetrahydro-5H-[1,2,3]triazolo [4,5-c]pyridin-5-yl)methanone (4.0 mg) in anhydrous DMSO (0.7 mL) was added,and the mixture was heated at 120°C for 15 min. Following ^18^F-fluorination,the crude reaction mixture was directly transferred to a stainless-steel loop and injected into a high-performance liquid chromatography (HPLC) system with a semipreparative column (Phenomenex Luna C18 10 × 250 mm,5 μm) as the stationary phase. A mixture of acetonitrile (ACN with 0.1% trifluoroacetic acid -TFA-)/(MQW with 0.1% TFA) (65/35) was used as the mobile phase under isocratic conditions with a 4 mL/min flow. The desired fraction (retention time = 18.5 min; [^18^F]JNJ-64413739) was collected into a separate vessel,diluted with a sodium ascorbate solution (3% w/v,25 mL) and reformulated via a C-18 light cartridge (Sep-Pak® Light,Waters,Milford,MA,USA). The cartridge containing the product was eluted with pure ethanol (1.0 mL) into the final vial,which was directly used for dose formulation. Chemical and radiochemical purities were determined via radio-HPLC (Agilent 1200 Series,USA) equipped with a radio-detector (Gabi,Elysia-Raytest,Germany) and a UV detector (Agilent,USA) connected in series. An Agilent Eclipse XBD-C18 (4.6 × 150 mm,5 μm) was used as the stationary phase,and a mixture of acetonitrile/water containing 0.1% trifluoroacetic acid (ACN 0.1% TFA)/(MQW 0.1% TFA) was used as the mobile phase under gradient conditions (0 min [5% ACN]; 0–2 min [5% ACN]; 2–20 min [from 5% to 95% ACN,linear]; 20–24 min [95% ACN]; 24–28 min [from 95% to 5% ACN,linear]) with a 1 mL/min flow. The identity of the product was confirmed via radio-HPLC (retention time = 12.5 min; [^18^F]JNJ-64413739). The radiochemical yield was 3.8% (decay-corrected to start the synthesis),with a radiochemical purity of 99% and a molar activity of 37.2 GBq/µmol at the end of the synthesis. The total synthesis time was 70 min.

**References**

1. Berdyyeva T,Xia C,Taylor N,He Y,Chen G,Huang C,Zhang W,Kolb H,Letavic M,Bhattacharya A,Szardenings AK (2019) PET Imaging of the P2X7 Ion Channel with a Novel Tracer [(18)F]JNJ-64413739 in a Rat Model of Neuroinflammation. Mol Imaging Biol.21(5):871-8.

2. Mathys H,Boix CA,Akay LA,Xia Z,Davila-Velderrain J,Ng AP,Jiang X,Abdelhady G,Galani K,Mantero J,Band N,James BT,Babu S,Galiana-Melendez F,Louderback K,Prokopenko D,Tanzi RE,Bennett DA,Tsai LH,Kellis M (2024) Single-cell multiregion dissection of Alzheimer's disease. Nature.
